# Supplementary material for: Genome and Genetic Engineering of the House Cricket (Acheta domesticus): A Resource for Sustainable Agriculture
Source: Biomolecules. 2023 Mar 24;13(4):589. doi: 10.3390/biom13040589 (PMC10136058; doi:10.3390/biom13040589)
Supplement: Supplementary file 1 [file biomolecules-13-00589-s001.zip › Supplementary_Materials/S12Fig.docx]

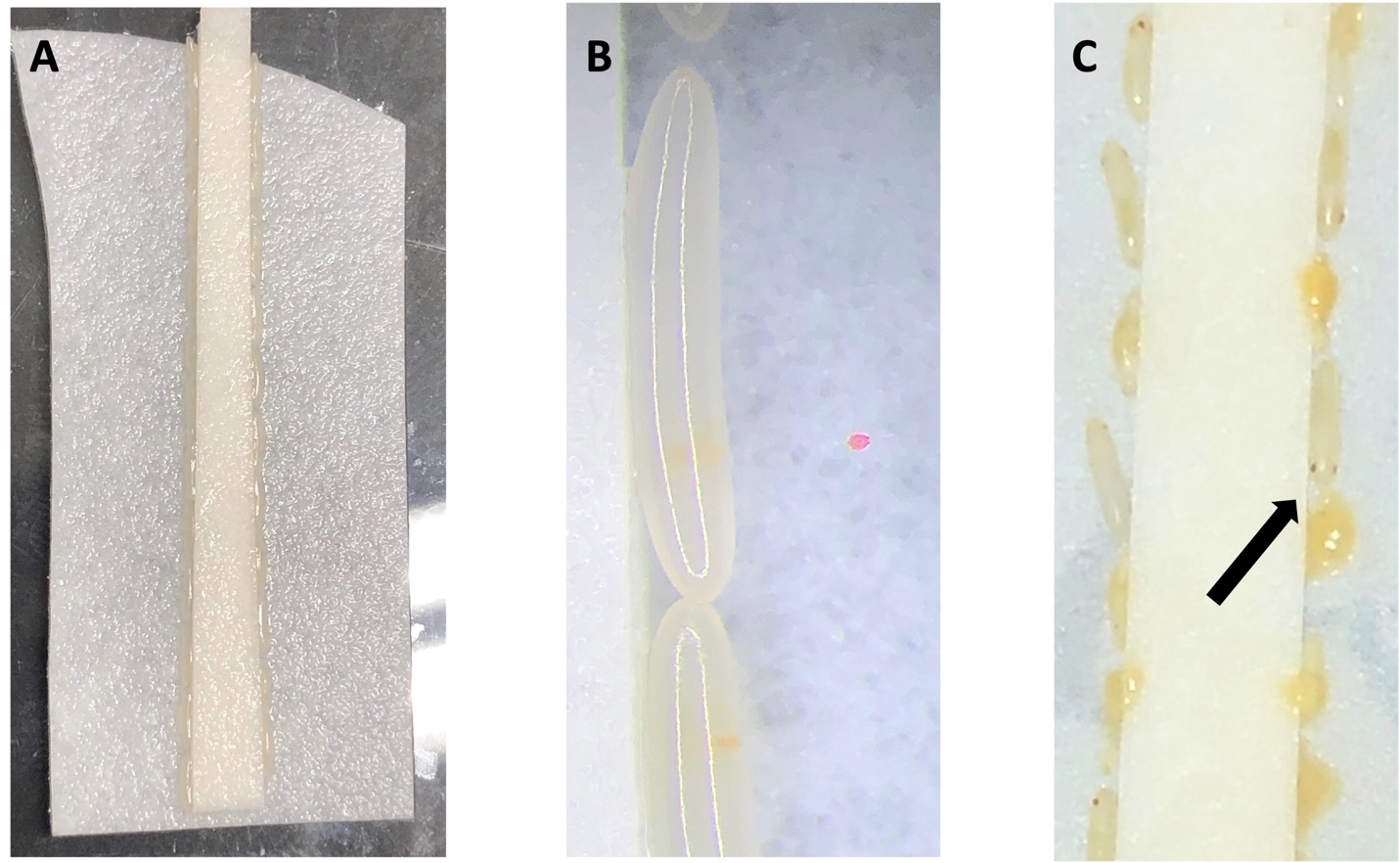


**Fig S12. *A. domesticus* egg microinjection slide.** A) Slide set up with eggs positioned on each side of the filter paper strips; B) Injected eggs showing microinjection site with phenol red color in eggs; and C) Developed eggs after microinjection showing eye pigmentation.
