# Supplementary material for: Genome and Genetic Engineering of the House Cricket (Acheta domesticus): A Resource for Sustainable Agriculture
Source: Biomolecules. 2023 Mar 24;13(4):589. doi: 10.3390/biom13040589 (PMC10136058; doi:10.3390/biom13040589)
Supplement: Supplementary file 1 [file biomolecules-13-00589-s001.zip › Supplementary_Materials/S1Table.docx]

**Table S1. Genome size of *A. domesticus****.* 1C = the amount of DNA in a gamete (1C is an average of the gametes with and without the X in the male).

| **Sex** | **1C (Mbp)** | **SE** | **N** |
| --- | --- | --- | --- |
| Female | 2,378.8 | 9.7 | 11 |
| Male | 2,149.6 | 11.2 | 12 |
