# Supplementary material for: Genome and Genetic Engineering of the House Cricket (Acheta domesticus): A Resource for Sustainable Agriculture
Source: Biomolecules. 2023 Mar 24;13(4):589. doi: 10.3390/biom13040589 (PMC10136058; doi:10.3390/biom13040589)
Supplement: Supplementary file 1 [file biomolecules-13-00589-s001.zip › Supplementary_Materials/S2Fig.docx]

**Fig S1. Comparison of the lengths of BUSCO reference genes (bp) in *Tribolium castaneum* and *Acheta domesticus.***
