# Supplementary material for: Genome and Genetic Engineering of the House Cricket (Acheta domesticus): A Resource for Sustainable Agriculture
Source: Biomolecules. 2023 Mar 24;13(4):589. doi: 10.3390/biom13040589 (PMC10136058; doi:10.3390/biom13040589)

Figure S6a

Correlation between total repeat content and genome size

w/ locust   w/o locust

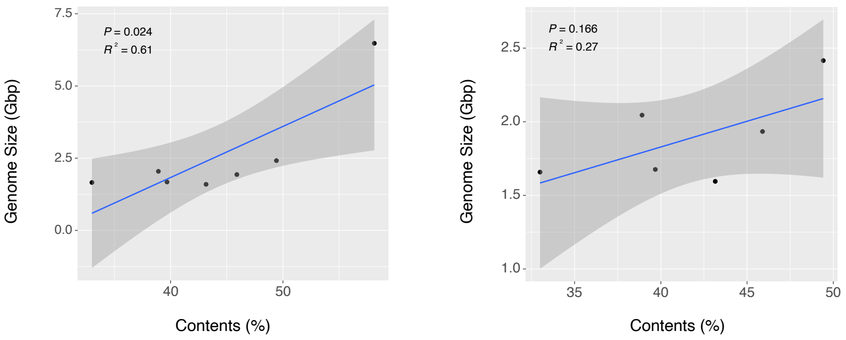

Correlation between major TEs contents and genome size in crickets (w/o locust)

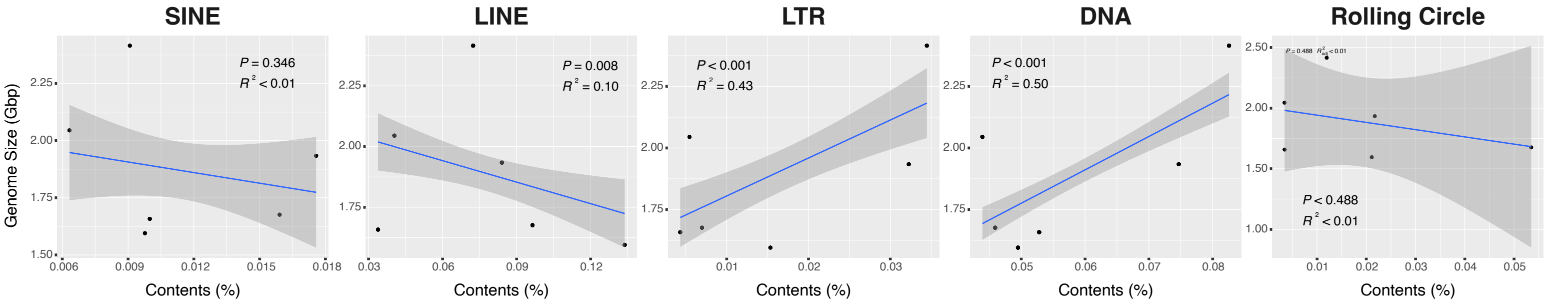

Figure S6b

Correlation between total repeat content and median length of intron

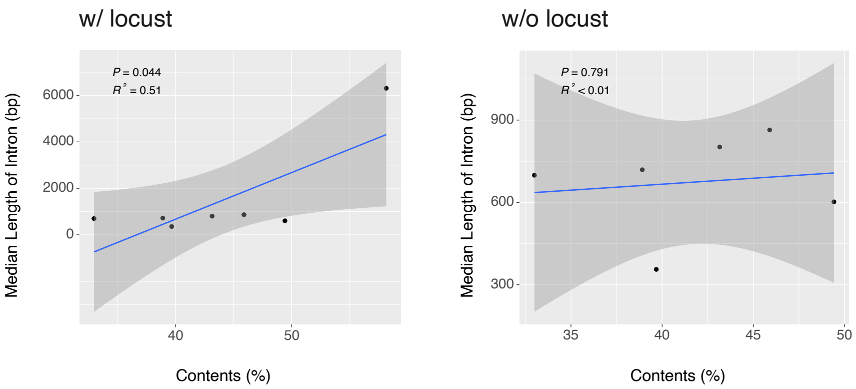

Correlation between major TEs contents and median length of intron (w/o locust)

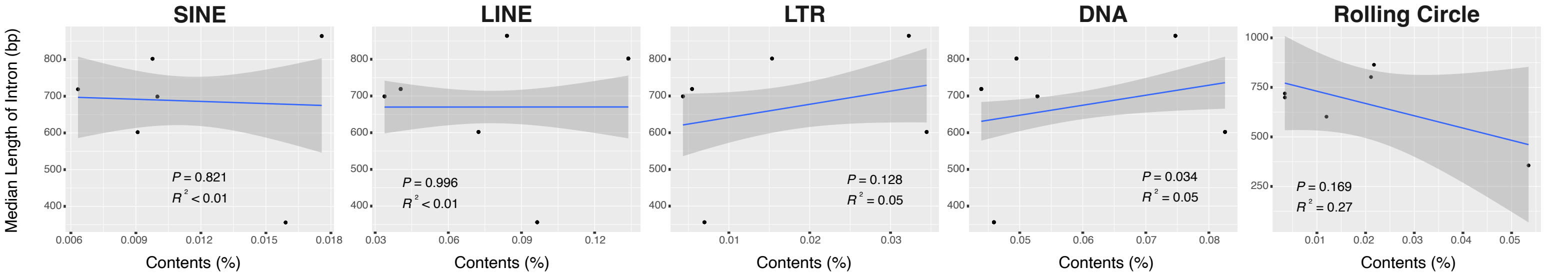

Figure S6c

Correlation between genome size and median length of intron

w/ locust

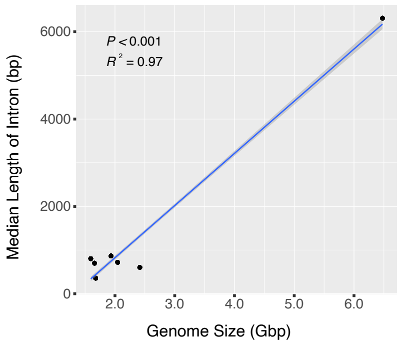

w/o locust

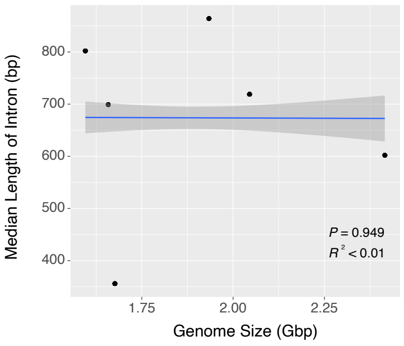

Figure S6d

LTR

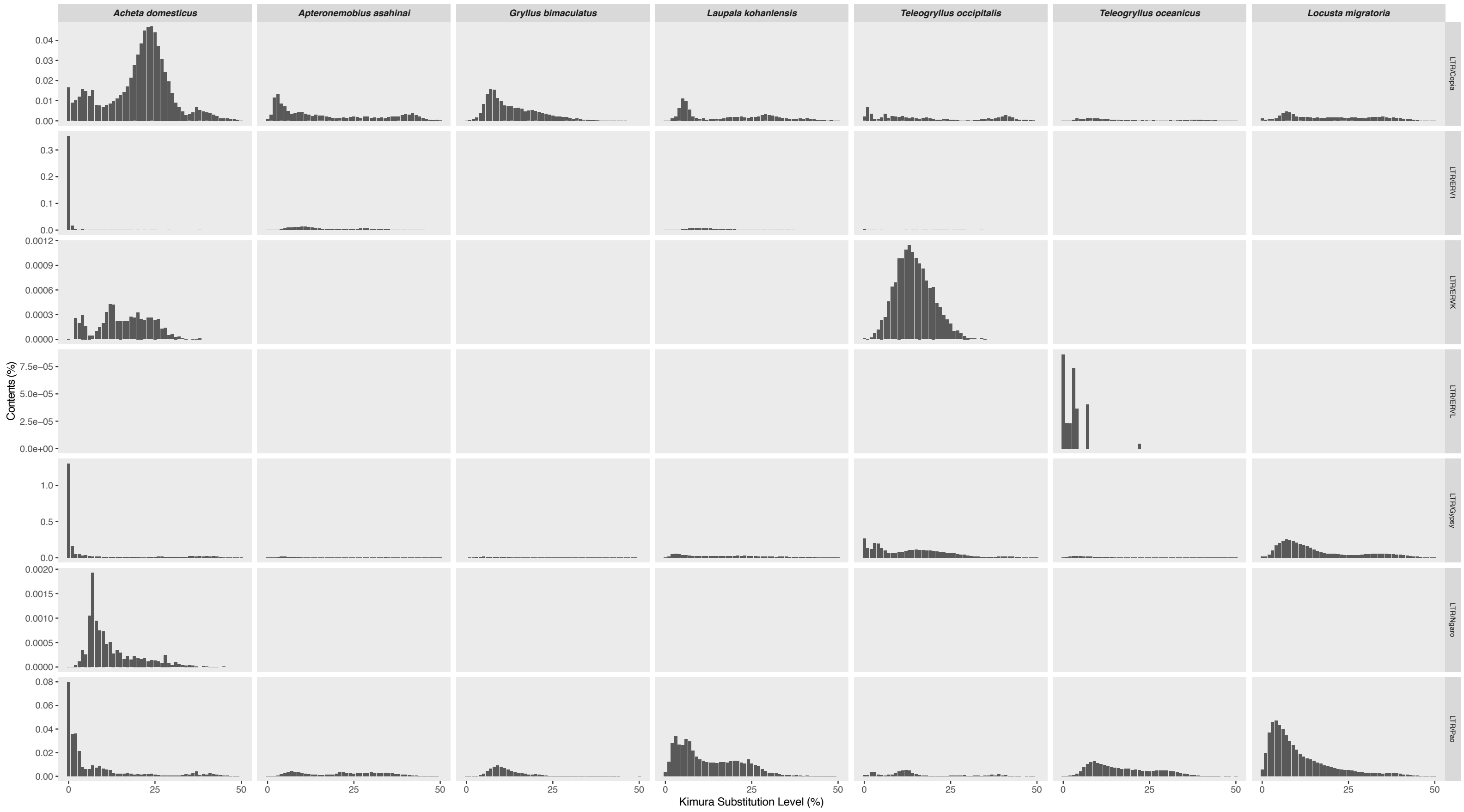

Figure S6e

LINE

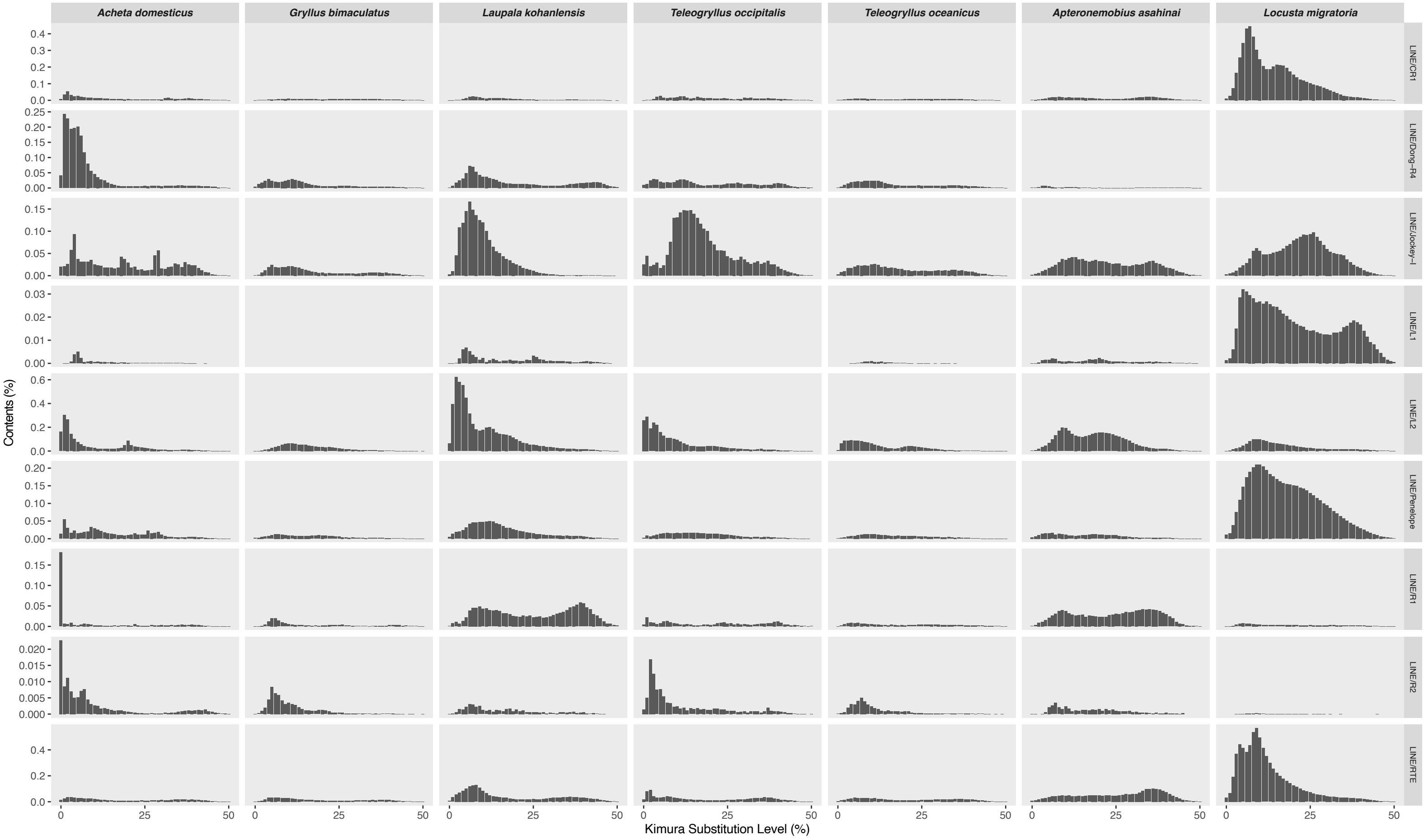

Figure S6f

DNA

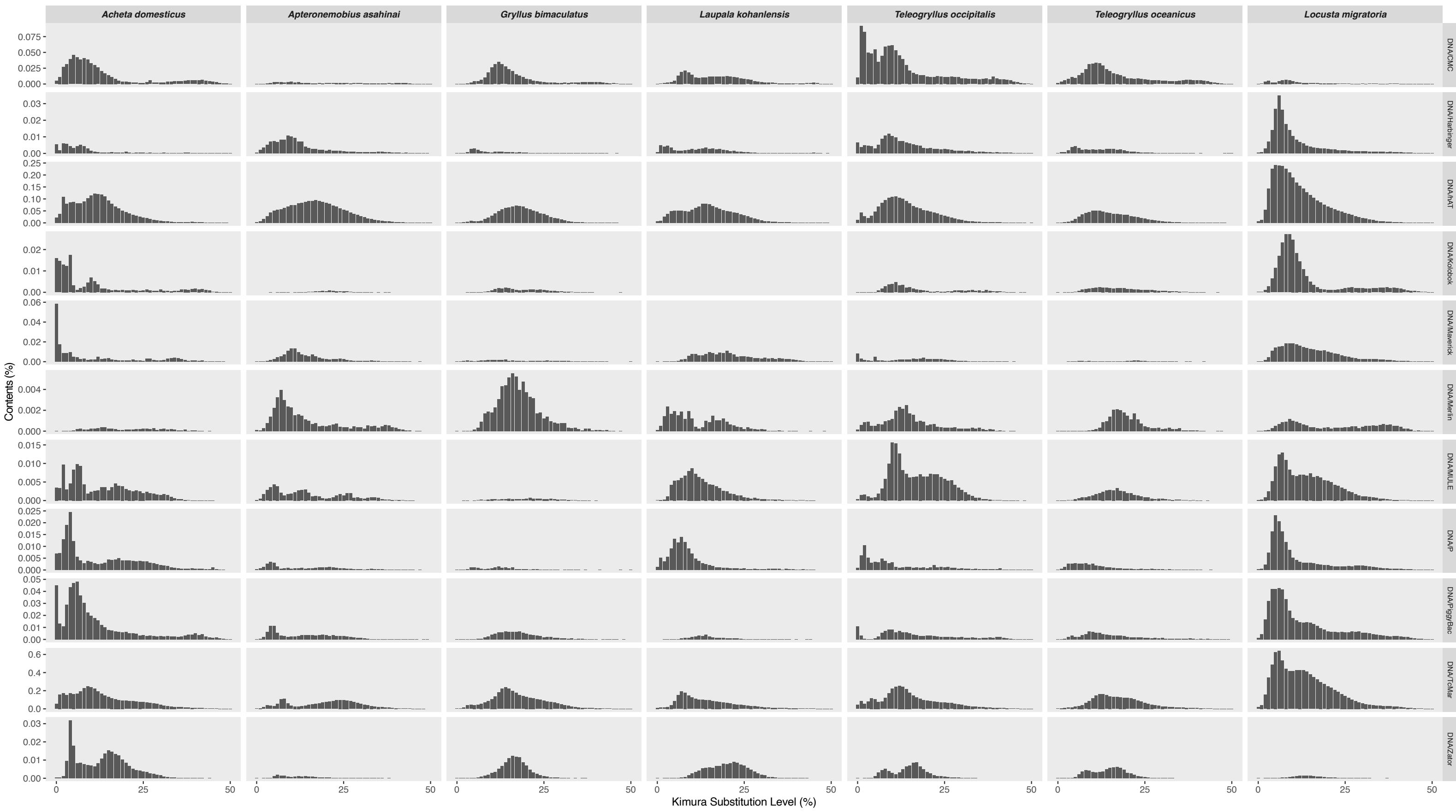

Supplement: Supplementary file 1 [file biomolecules-13-00589-s001.zip › Supplementary_Materials/S6Fig.pdf]
