# Supplementary material for: Genome and Genetic Engineering of the House Cricket (Acheta domesticus): A Resource for Sustainable Agriculture
Source: Biomolecules. 2023 Mar 24;13(4):589. doi: 10.3390/biom13040589 (PMC10136058; doi:10.3390/biom13040589)
Supplement: Supplementary file 1 [file biomolecules-13-00589-s001.zip › Supplementary_Materials/S8Tabledocx.docx]

**Table S8.** **CRISPR sgRNA target sequences for the *Ad vermillion* gene.**

| **Name** | **Target sequence** | **PAN** |
| --- | --- | --- |
| AdV sgRNA #1 | GAGGCGCCGGGCGCTCAGGA | CGG |
| AdV sgRNA #2 | GAGGTGCTGGACGGGAACGT | GGG |
| AdV sgRNA #3 | GTACGCCGACTACCTGCAGC | TGG |
